# Develop a High-Throughput Screening Method to Identify C-P4H1 (Collagen Prolyl 4-Hydroxylase 1) Inhibitors from FDA-Approved Chemicals

**DOI:** 10.3390/ijms21186613

**Published:** 2020-09-10

**Authors:** Shike Wang, Kuo-Hao Lee, Nathalia Victoria Araujo, Chang-Guo Zhan, Vivek M. Rangnekar, Ren Xu

**Affiliations:** 1 Markey Cancer Center, University of Kentucky, Lexington, KY 40536, USA; shike.wang@uky.edu; 2 Department of Pharmacology and Nutritional Sciences, University of Kentucky, 789 S. Limestone Rd., Lexington, KY 40536, USA; 3 Pharmaceutical Sciences, University of Kentucky, Lexington, KY 40536, USA; kuohaolee@uky.edu (K.-H.L.); chang-guo.zhan@uky.edu (C.-G.Z.); 4 Department of Toxicology and Cancer Biology, University of Kentucky, Lexington, KY 40536, USA; nathalia.vitoria.araujo@uky.edu (N.V.A.); vmrang01@uky.edu (V.M.R.); 5 Radiation Medicine, University of Kentucky, Lexington, KY 40536, USA

**Keywords:** prolyl hydroxylation, collagen, high-throughput screening, extracellular matrix, cancer invasion

## Abstract

Collagen prolyl 4-hydroxylase 1 (C-P4H1) is an α-ketoglutarate (α-KG)-dependent dioxygenase that catalyzes 4-hydroxylation of proline on collagen. C-P4H1-induced prolyl hydroxylation is required for proper collagen deposition and cancer metastasis. Therefore, targeting C-P4H1 is considered a potential therapeutic strategy for collagen-related cancer progression and metastasis. However, no C-P4H1 inhibitors are available for clinical testing, and the high content assay is currently not available for C-P4H1 inhibitor screening. In the present study, we developed a high-throughput screening assay by quantifying succinate, a byproduct of C-P4H-catalyzed hydroxylation. C-P4H1 is the major isoform of collagen prolyl 4-hydroxylases (CP4Hs) that contributes the majority prolyl 4-hydroxylase activity. Using C-P4H1 tetramer purified from the eukaryotic expression system, we showed that the Succinate-GloTM Hydroxylase assay was more sensitive for measuring C-P4H1 activity compared with the hydroxyproline colorimetric assay. Next, we performed high-throughput screening with the FDA-approved drug library and identified several new C-P4H1 inhibitors, including Silodosin and Ticlopidine. Silodosin and Ticlopidine inhibited C-P4H1 activity in a dose-dependent manner and suppressed collagen secretion and tumor invasion in 3D tissue culture. These C-P4H1 inhibitors provide new agents to test clinical potential of targeting C-P4H1 in suppressing cancer progression and metastasis.

## 1. Introduction

Collagen is the most abundant extracellular matrix (ECM) protein in the human body [1]. Increased collagen expression and deposition is associated with fibrosis and tumor progression, and reducing collagen production is sufficient to inhibit the progression of these diseases [2,3,4]. Collagen is synthesized and forms the triple helix structure in the ER [5]. The basic unit of the triple-helical structure is Gly-pro-X, and the proline is often hydroxylated. Prolyl hydroxylation is required for the sharp twisting of collagen helix and secretion of collagen protein [6]. Therefore, the proline hydroxylation pathway is considered a promising target to halt collagen expression and deposition.

Collagen hydroxylation is catalyzed by collagen prolyl 4-hydroxylase (C-P4H) [7], a Fe^2+^ and 2-oxoglutarate-dependent enzyme [7]. C-P4H contains two α subunits (P4HA) and two β subunits (P4HB), and each α subunit contains the peptide substrate binding domain and the catalytic domain [8]. Three P4HA isoforms have been characterized in mammalian cells with different tissue distribution. It has been shown that P4HA1 contributes more than 50% of prolyl 4-hydroxylase activity in most cell types and considered the major isoform [9].

Increased collagen expression or deposition is associated with cancer development, such as gastric cancer [10], breast cancer [11], bladder cancer [12], and colorectal cancer [13]. Binding of collagen to its receptors such as discoidin domain receptors (DDRs) [14] and integrin [15,16] activates downstream signal pathways, and subsequently enhances cancer cell migration and invasion [17,18]. It has been shown that P4HA1 expression is induced during cancer development and progression. Silence of P4HA1 is sufficient to inhibit cancer metastasis and sensitize cancer cells to chemotherapeutic agents [19]. Increased P4HA1 expression has also been detected in fibrosis, and inhibition of P4HA1 reduces hepatic collagen accumulation and suppresses the fibrosis progression [20]. Overexpression of P4HA1 increases atherosclerotic plaque in apolipoprotein E-deficient mice, suggesting a potential role of P4HA1 in coronary thrombosis [21]. These results suggest that P4HA1 is a potential therapeutic target for cancer and fibrosis.

A HPLC-based assay has been developed to measure C-P4H1 activity based on P4H-catalyzed turnover of a flp-containing peptide [22]. This assay has been used to examine small molecule activities in the small scale, resulting in the identification of several chemicals as C-P4H1 inhibitor, including ethyl-3,4-dihydroxybenzoic acid (DHB) and 2,2′-bipyridine (bipy) [23,24]. Although their inhibitory activities on collagen secretion has been confirmed in tissue culture, none of them has been approved for clinical use. There is an urgent need to identify potent C-P4H1 inhibitors that can be used in clinic for the treatment of collagen-dependent diseases.

Here, we described a novel method to measure C-P4H1 activity by quantifying the product of prolyl hydroxylation, succinate. We developed a high-throughput screening assay based on this method and identified Silodosin and Ticlopidine as novel C-P4H1 inhibitors from the FDA-approved drug library. Further examining their biological activity in vivo may identify Silodosin and Ticlopidine as potential drugs targeting C-P4H1-dependent cancer progression.

## 2. Results

### 2.1. Protein Expression and Purification

**Figure 1:**
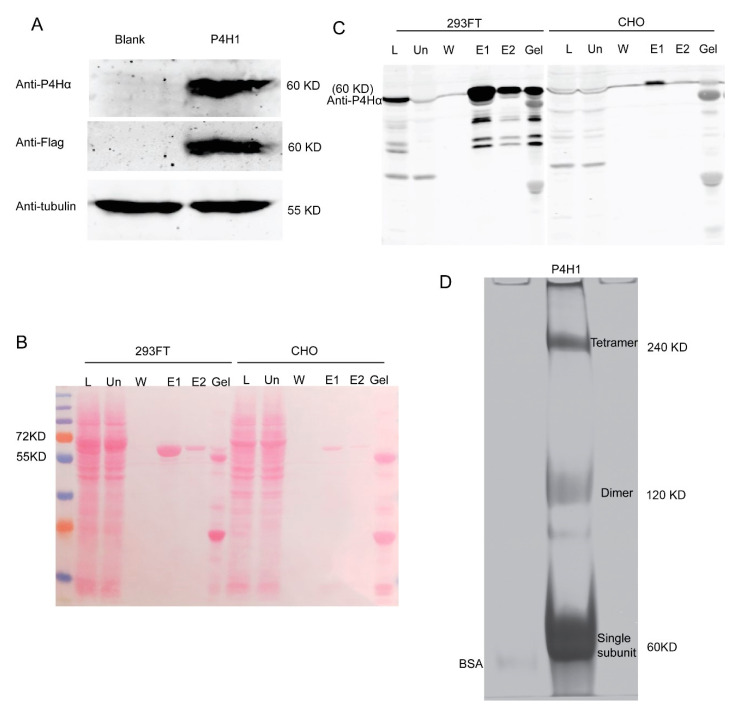
C-P4H1 is expressed and purified from HEK-293FT cells. (**A**) Expression of C-P4H1 was analyzed by western blot with anti-P4H1α and anti-flag antibody. Cell lysates were collected from control and 293 FT cells transfected with P4HA1 and P4HB constructs. (**B**) Ponceau staining showed the expression and purification of C-P4H1 from 293 FT cells and CHO cells. The letters indicate: L (Total cell lysates with HGLB), Un (unbinding samples after cell lysates incubated with M2 gel), W (The last time washed sample in NET2), E1 (The first time elution sample in 0.25 µg/µL 3× Flag), E2 (The second time elution sample in 0.25 µg/µL 3× Flag), Gel (The remaining sample on M2 gel after elution). (**C**) Western blot analysis of C-P4H1 expression and purification with anti-P4Hα. (**D**) Purified P4H1 samples was analyzed by 8% Native PAGE gel with Coomassie Blue staining; BSA was included as a control.

C-P4H1 contains two α1 subunits and two β subunits. The β subunit is the protein disulfide isomerase (PDI) [25], which is required for catalytic activity of the α subunit [26]. Therefore, it is necessary to purify the C-P4H1 tetramer for measuring its activity. In previous studies, C-P4Hs were often expressed and purified from *E. coli* [27]. Given the important function of post modification for protein activity, we decided to use mammalian cell lines for C-P4H1 expression. HEK-293 FT and CHO have been widely used to expression exogenous proteins with high transfection efficiency [28]. P4HA1 and P4HB expression constructs with flag tag were transfected into HEK-293FT cells. The cells were harvested 48 h after transfection, and P4HA1 expression was examined by western blotting with antibodies against P4HA1 and Flag (Figure 1A).

Next, we compared C-P4H1 expression in HEK-293FT cells and CHO cells. P4HA1 and P4HB expression constructs were transfected into these two cell lines, and the recombinant C-P4H1 was purified with anti-Flag M2 beads. We found that the P4H α1 was expressed and purified at much higher levels in HEK-293FT cells than in CHO cells (Figure 1B,C). Therefore, HEK-293FT cells were used to generate C-P4H1 for the following experiments.

To determine whether the C-P4H1 tetramer was purified with anti-Flag M2 beads, we performed native gel electrophoresis to analyze the purified protein. Coomassie blue staining results showed that the purified protein presented at three major bands, and tetramer, dimer and single subunit were all detected (Figure 1D).

### 2.2. Screening Method Confirmation

**Figure 2:**
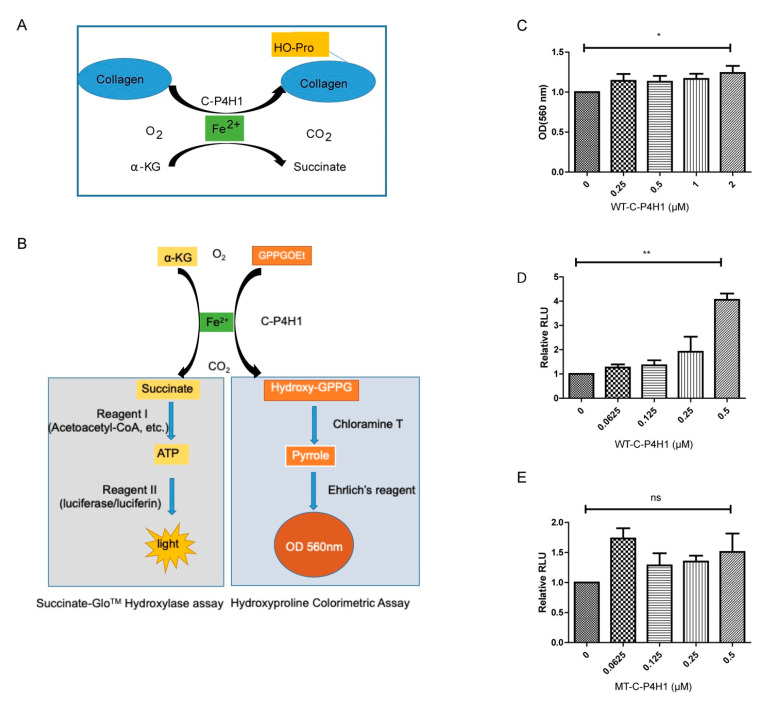
Hydroxyproline Colorimetric Assay and Succinate-GloTM Hydroxylase assay are developed to analyze C-P4H1 activity. (**A**) A scheme showing collagen hydroxyproline reaction. (**B**) A scheme showed how the Succinate-GloTM Hydroxylase assay (Left) which was developed to detect succinate, and how Hydroxyproline Colorimetric Assay (Right) was used to detect the HO-GPPG. (**C**) C-P4H1 activity was evaluated with the Hydroxyproline Colorimetric Assay at different concentrations. *n* = 3. * *p* value < 0.05; one-way ANOVA analysis. (**D**) C-P4H1 activity was measured with the Succinate-GloTM Hydroxylase assay at different concentration of protein. *n* = 3. ** *p* value < 0.01. One-way ANOVA analysis. (**E**) MT-P4HA1 activity was evaluated with the Succinate-GloTM Hydroxylase assay at different concentrations. *n* = 3. ns, no statistical significance. The data displayed as means ± standard deviation (SD).

The colorimetric assay has been used to evaluate hydroxyproline and quantified collagen levels in ECM [29,30,31], in which hydroxyproline reacts with p-dimethylaminobenzaldehyde (DMAB, Ehrlich’s reagent) to produce the chromophore (Figure 2B). However, this assay has not been used to characterize C-P4Hs inhibitors. Bioluminescence-based Succinate-GloTM Hydroxylase assay (Figure 2B) has been used to measure protein hydroxylase activity with the high content potential [32]. We compared these two assays with different concentration of C-P4H1. The OD_560_ value in the hydroxyproline reaction was moderately increased at 0.25 µM C-P4H1 compared to negative control, and further increasing the concentration of C-P4H1 had little effect on the OD value (Figure 2C). Luminescence values in the Succinate-Glo™ assay were induced by C-P4H1 in a dose-dependent manner, and two-fold induction was detected at 0.25 µM of C-P4H1(Figure 2D). These results indicate that the bioluminescence-based Succinate-Glo™ assay is more sensitive for evaluating C-P4H1 activity than the colorimetric assay.

It has been shown that mutation of P4HA1 H483 abolished the prolyl hydroxylase activity without any impact on tetramer formation [33]. We cloned the mutant P4HA1 H483S into pcDNA 3.1 vector. The mutant protein (MT-C-P4H1) was expressed in HEK-293FT cells and purified using the same protocol with the wild type C-P4H1 (WT-C-P4H1). We found that the mutant C-P4H1 failed to increase the luminescence values in the Succinate-Glo™ assay (Figure 2E). These results indicate that Succinate-Glo™ assay specifically detects prolyl hydroxylase activity of C-P4H1.

**Figure 3:**
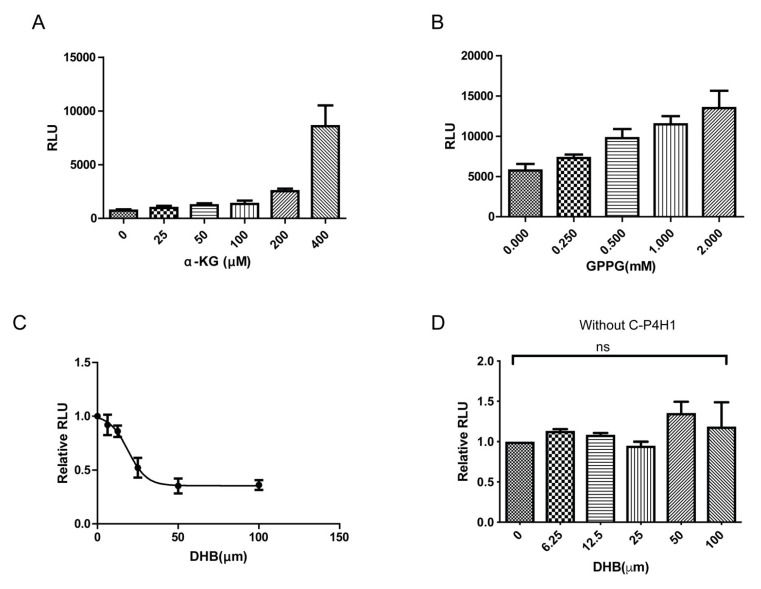
Evaluation of the Succinate-GloTM Hydroxylase assay for analyzing C-P4H1 activity. (**A**) Bar graph showing the C-P4H1 (200 nM) activity at different concentrations of 2-KG. *n* = 3. (**B**) Bar graph showing the C-P4H1 (200 nM) activity at different concentrations of substrate peptide GPPGOET. *n* = 3. (**C**) Curve measurement of the inhibitory activity of DHB in C-P4H1 (200 nM) reaction. All the ATP signals produced by the plate reader were normalized with control. *n* = 3. (**D**) DHB had little effect on the Succinate-GloTM Hydroxylase assay in the absence of C-P4H1. All the ATP signals produced by the plate reader were normalized with control, control value is 1. *n* = 3. ns means no significant statistic. The data displayed as means ± standard deviation (SD).

To further evaluate reliability of the Succinate-Glo™ assay, we tested varied concentration of peptide substrate GPPG (Figure 3A) and hydroxyl donor α-KG (Figure 3B) in the hydroxylation reaction, respectively. We found that increased concentration of GPPG and α-KG induced luminescence assay in a dose-dependent manner. DHB has been identified as a C-P4Hs’ inhibitor [34]. To determine whether this assay can evaluate C-P4H1 inhibitor’s activity, C-P4H1 was incubated with different concentration of DHB for 30 min at 4 °C, and then the hydroxylation reaction and the bioluminescence-based assay were performed. Treatment with DHB significantly reduced luminescence values (Figure 3C). The IC50 was detected at around 20 µM, which is much lower than the IC50 value of 0.1 mM reported in the previous research with the collagen hydroxylation assay in the chicken tendon cells [35]; this is probably due to the sensitivity difference between two methods. In the absence of C-P4H1, DHB had little effect on the Succinate-Glo™ assay, indicating the inhibitory effect is specific on the prolyl hydroxylation reaction (Figure 3D). These results demonstrate that the bioluminescence-based Succinate-GloTM Hydroxylase assay can be used to screen C-P4H1 inhibitors.

### 2.3. Inhibitory Screening and Verification

**Figure 4:**
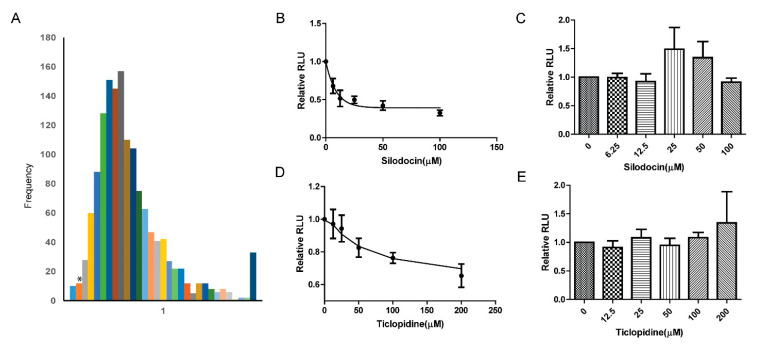
C-P4H1 inhibitors are identified with the high-throughput screening assay from the FDA-approved drug library. (**A**) The frequency for relative activity of P4H1 at the presence of each chemical. Over 1400 FDA-approved drugs were screening in the 384 well plate or 96-well plate at 50 µM. 50 µM DHB was used as a positive control in each plate. All the ATP signals produced by the plate reader were normalized with the equation: $relative\;activity=\frac{value\;of\;samples\;with\;chemicals}{value\;of\;control\;sample\;without\;chemicals}$. *, the relative activity of C-P4H1 in the presence of DHB. (**B**) Silodocin inhibitory activity was analyzed at different concentrations. All the ATP signals produced by the plate reader were normalized to vehicle control, control value is 1; *n* = 3. (**C**) Silodocin did not inhibit the Succinate-GloTM Hydroxylase assay in the absence of WT-P4H1; *n* = 3. (**D**) Ticlopidine inhibitory activity was analyzed at different concentrations; 200 nM C-P4H1 in the reaction. The C-P4H1 activities in ticlopidine-treated samples were normalized with vehicle control, control value is 1; *n* = 3. (**E**) Ticlopidine did not inhibit the Succinate-GloTM Hydroxylase assay in the absence of WT-P4H1; *n* = 3. The data displayed as means ± standard deviation (SD).

Repurposing FDA-approved drugs is a popular strategy to facilitate clinical studies of new therapeutic targets [36]. To identify new C-P4H1 inhibitors that can be tested in clinic, we performed a moderate scale screening with the bioluminescence-based assay using more than 1400 FDA-approved chemicals. We identified over 40 chemicals (Figure 4A) with greater or similar inhibitory ability compared to DHB. We further tested the inhibitory activities of two drugs, Silodosin and Ticlopidine, at different concentration. Both drugs inhibited C-P4H1 activity in a dose-dependent manner (Figure 4B,D). Interestingly, Silodosin showed a faster kinetic property compared to ticlopidine, suggesting the different chemical-protein interaction mechanism between two drugs. The two chemicals had little effect on the luminescence assay in the absence of C-P4H1 (Figure 4C,E), indicating that Silodosin and Ticlopidine specifically inhibited the C-P4H1 activity in vitro.

**Figure 5:**
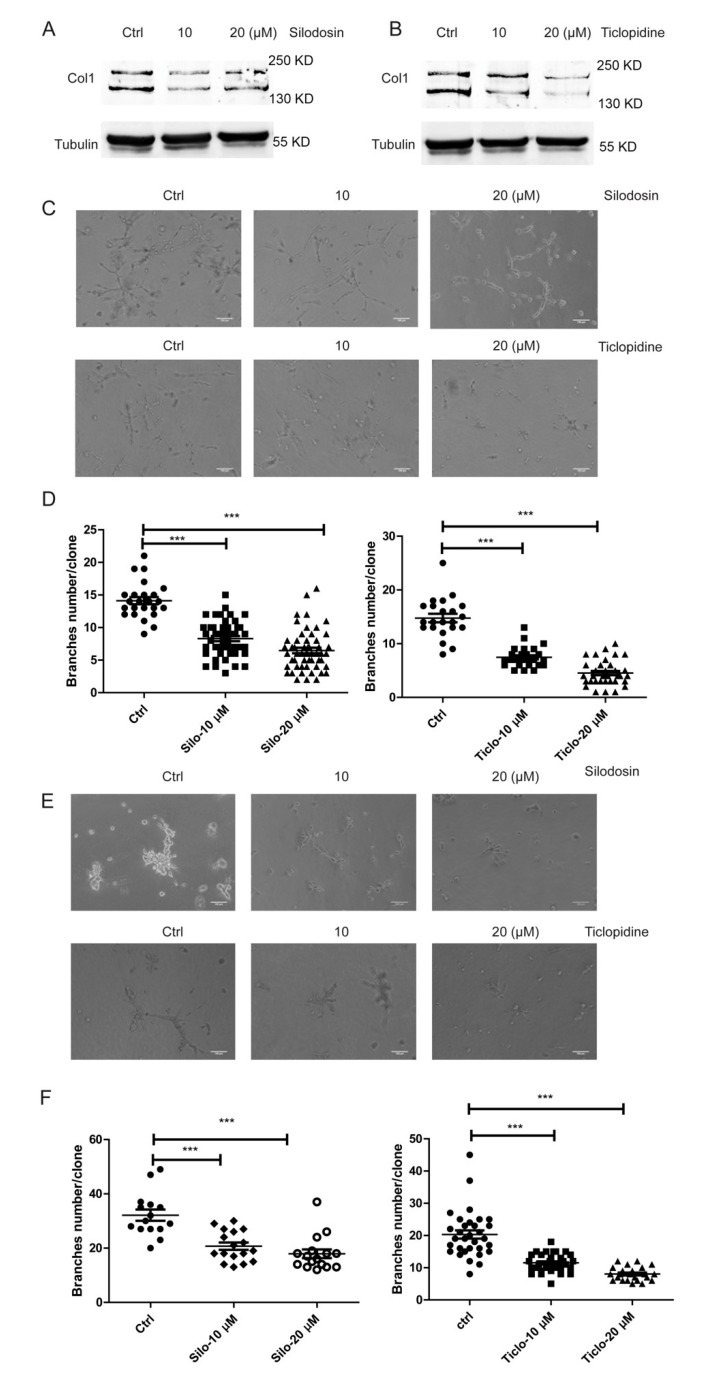
Silodocin and ticlopidine inhibit collagen deposition and cancer cell invasion. (**A**) Western blot analyzed levels of type I collagen in conditional medium of HS578 cells, The cells were treated with Silodosin at 10 µM and 20 µM. (**B**) Western blot analyzed levels of type I collagen in conditional medium of HS578 cells, The cells were treated with Ticlopidine at 10 µM and 20 µM. (**C**) Invasiveness of MDA-MB-231 cells was examined in 3D culture assay in the presence or absence of Silodosin and Ticlopidine. The cells were with Silodosin or Ticlopidine at 10 µM and 20 µM, and images were taken after 48 h. Scale bar, 100 µm. (**D**) Dot blot graph quantified invasive branching structures in panel C. One-way ANOVA analysis; *n* ≥ 20. *** *p* ≤ 0.001. (**E**) Phase images of control and Hs-578 cells treated with Silodosin and Ticlopidine at 10 µM and 20 µM in 3D culture assay. Images were taken at 24 h after treatment. Scale bar, 100 µm. (**F**) Dot blot graph quantified invasive branching structures in panel E. One-way ANOVA analysis; *n* ≥ 20. *** *p* ≤ 0.001.

To determine whether the identified potential inhibitors suppress the C-P4H1 activity in tissue culture, we treated HS-578T cells with Silodosin or Ticlopidine for 48 h. Since collagen maturation and secretion was regulated by the C-P4H1, conditioned media were collected from control and Silodosin- or Ticlopidine-treated cells for western blot analysis. We found that treatment with Silodosin or Ticlopidine reduced protein levels of type I collagen in the conditioned media (Figure 5A,B). These results suggest that Silodosin and Ticlopidine can inhibit C-P4H1 activity in vivo.

It has been shown that silence of P4HAs suppress invasive tumor growth in three-dimensional (3D) tissue culture model [19,37]. 3D culture model has been widely used to evaluate drug effect [38]. MDA-MB-231 cells and HS-578T cells are triple-negative breast cancer cell lines with invasive phenotypes in 3D culture model [39]. To further evaluate the inhibitory ability of these chemicals in tumor progression, we treated these two cell lines with Silodosin and Ticlopidine in 3D culture. Both chemicals significantly reduced invasive branching structures of MDA-MB-231 cells (Figure 5C,D) and HS-578T cells (Figure 5E,F). Invasive branching structure of cancer cells in 3D culture is associated with tumor invasion and aggressive phenotypes [15,19,37]. We and others showed previously that knocking down P4HA1 inhibited invasive branching in triple-negative breast cancer cells but had very little effect on cell proliferation [19,37]. To test whether Silodosin or Ticlopidine treatment inhibits tumor cell proliferation, we incubated the MDA-MB-231 and HS-578T cells with these two drugs and analyzed cell number and viability with CCK-8 kit. We found that treatment with Silodosin or Ticlopidine had little effect on cell proliferation in 2D culture (Data are not shown). These results demonstrate that the C-P4H1 inhibitors identified from the Succinate-Glo™ assay suppressed invasive phenotypes of triple-negative breast cancer cells.

## 3. Discussion

In the present study, we developed a new method to quantify the C-P4H1 activity by measuring succinate levels. Using the high-throughput screening assay based on this method, we identified several new C-P4H1 inhibitors, including Silodosin and Ticlopidine, from the FDA-approved drug library. We further confirmed that treatment with Silodosin and Ticlopidine reduced collagen secretion and suppressed invasive phenotypes of breast cancer cells in 3D culture.

To develop a better method to quantify the C-P4H1 activity, we compared the hydroxyproline colorimetric assay to measure hydroxyproline and Succinate-Glo^TM^ Hydroxylase assay to measure succinate levels. We found that the latter is more sensitive for measuring the C-P4H1 activity. One potential reason for the reduced sensitivity in the hydroxylation colorimetric assay is the short peptide length and Pro position of the substrate compared to collagen protein [40]. Thus, using longer peptide as a substrate may increase the hydroxylation reaction rate and improve the colorimetric assay sensitivity. As mentioned above, HPLC has been used to identify small molecules that inhibit the C-P4H1 activity. Compared with the HPLC-based assay, the method that we developed with Succinate-Glo^TM^ Hydroxylase assay has the potential for large scale screening.

Using the Succinate-Glo^TM^ Hydroxylase assay, we identified more than 40 potential C-P4H1 inhibitors from the FDA-approved drug library. These inhibitors have been used for the treatment of various diseases, but most of them have not been tested in collagen-related diseases. Interestingly, we also found that several chemicals enhanced the enzyme activity, indicating that this method can be used to identify potential C-P4H1 activators. Collagen deficiency is associated with tissue adhesion diseases, including Ehlers–Danlos Syndrome and Osteoporosis [41]. Enhancing collagen producing by inducing C-P4H1 activity is a potential strategy for the treatment of these diseases.

One limitation for the method that we developed is that the Succinate-Glo^TM^ Hydroxylase assay does not directly measure C-P4H1 activity. Chemicals that affect the conversion of succinate to ATP and luciferase reaction may be falsely identified from screening; therefore, the potential inhibitors and activators identified from the screening needs to be further verified with the Succinate-Glo^TM^ Hydroxylase assay in the absence of C-P4H1.

Most of the identified C-P4H1 inhibitors works as α-KG mimics or Fe^2+^ chelating agents [23]. It is not clear whether the inhibitors that we identified function through the same mechanism; therefore, it is important to compare the molecular structure of these inhibitors with those previously identified and determine how these inhibitors interacted with C-P4H1 in the future. The Quantitative structure-activity relationships (QSAR) play important role in drug design and structure optimization [42,43]. We also plan to perform the QSAR analysis to identify more potent C-P4H1 inhibitors.

In summary, we identified several C-P4H1 inhibitors from the FDA-approved drug library using the Succinate-Glo^TM^ Hydroxylase assay. We showed that two of them, Silodosin and Ticlopidine, inhibited collagen secretion and invasive tumor growth in tissue culture model. Further evaluating their activity in vivo may identify new drugs to target C-P4H1 induced cancer progression.

## 4. Martials and Methods

### 4.1. Cell Culture and 3D Culture Assay

MDA-MB-231 cells (ATCC) were cultured in DMEM/F12 (Sigma, St. Louis, MO, USA) with 10% fetal bovine serum (Invitrogen, Carlsbad, CA, USA). HS-578 (ATCC) cells were cultured in DMEM (Sigma) with 10% fetal bovine serum. 3D laminin-rich extracellular matrix (3D lrECM) on-top cultures were prepared by trypsinization of cells from tissue culture plastic [37]. Growth Factor Reduced BD Matrigel™ was plated on the bottom of the cell culture dish. MDA-MB-231 and HS-578 cells were seeded on the top of the Matrigel layer, and additional medium containing 10% Matrigel was added on the top. The cells lines were maintained below 10 passages.

### 4.2. Cell Viability Assay

To analyze cell viability in response to drug treatment, MDA-MB-231 and HS-578 cells were seeded into 96-well plate with the density of 3000 cells per well. Following the cell adhesion, chemicals were added and treated for 48 h. The cell viability was measured by the cell counting kit-8 (Sigma).

### 4.3. CP4H1 Expression and Purification

The CP4Hα and CP4Hβ gene were amplified with flag sequence then cloned into pcDNA 3.1 vector, respectively. Following primers were used for cloning; CP4Hα-N: AACGCTAGCGCCACCATGATCTGGTATATATTAAT; CP4Hα-C: ACTGGGATCCTCACTTATCGTCGTCATCCTTGTAATCTTCCAATTCTGACAACGTAC; CP4Hβ-N: AACGCTAGCGCCACCATGCTGCGCCGCGCTCTGCT; CP4Hβ-C: ACTGGGATCCTTACTTATCGTCGTCATCCTTGTAATCCAGTTCATCTTTCACAGCTT. pcDNA 3.1-CP4Hα-flag and pcDNA 3.1-CP4Hβ-flag plasmids were transfected into 293 FT cells with the Fugene reagent (Promega, Madison, WI, USA). Following 48 h’ transfection, cells were washed by phosphate-buffered saline (PBS) and lysed with HGLB (10 mM Tris-HCl pH 7.5, 10 mM NaCl, 2 mM EDTA, 0.5% Triton X-100) containing cocktail proteinase inhibitors (Sigma). Lysates were incubated with M2 gel (Sigma) at cold room for at least4 h, washed with NET2 buffer, and then eluted with 250 µg/mL 3× flag peptide (GP10149). The solution containing CP4H1 was concentrated with centrifugal filters (Sigma) and replaced with protein storing buffer (150 mM NaCl, 50 mM HEPES pH 7.4). The purified CP4H1 was subjected to native gel or SDS-PAGE gel.

### 4.4. Western Blot

Chemical treated Hs578 cells were lysed in 2% sodium dodecyl sulfate (SDS) in PBS buffer containing phosphatase and protease inhibitor cocktails (Sigma). Protein concentration was measured using Pierce™ BCA Protein Assay Kit (Thermo Fisher Scientific, Waltham, MA, USA). Equal amounts of protein lysates or cell conditional medium (normalized to cell numbers) were subjected to SDS gel electrophoresis, immunoblotted with primary antibodies (Anti-Collagen I antibody, ab34710; Anti-tubulin, Cell Signaling 2148)) and DyLight 680/800-conjugated secondary antibodies (DyLight 680-conjugated goat anti-rabbit IgG secondary antibody Thermo Fisher Scientific, 35569; DyLight 800-conjugated goat anti-mouse IgG secondary antibody Thermo Fisher Scientific, SA5-35521).

### 4.5. Measuring Human CP4H1 Activity with Hydroxyproline Colorimetric Assay and Succinate-Glo^TM^ Hydroxylase Assay

#### 4.5.1. Human CP4H1 Activity Assay

Human CP4H1 activity assays were carried out at room temperature in 10 mM HEPES buffer containing 10 mM NaCl, pH 7.4. Mix with human CP4H1 (200 nM), peptide substrate (GlyProProGlyOEt, 500 μM), FeSO_4_ (50 μM), catalase (0.1 mg/mL), ascorbate (2 mM), and α-ketoglutarate (100 μM). FeSO_4_ was prepared freshly in 10 mM HCl prior to each time use. Following one-hour reaction, the reaction solution was subjected to Hydroxyproline Colorimetric Assay and Succinate-GloTM Hydroxylase assay, respectively.

#### 4.5.2. Hydroxyproline Colorimetric Assay

100 µL of the Chloramine-T reagent (0.282 g chloramine-T, 1 mL n-propanol, 1 mL demineralized water, and 8 mL stock buffer, stock buffer consisting of 0.24 M citric acid, 0.88 M sodium acetate trihydrate, 0.88 M anhydrous sodium acetate, 0.21 M acetic acid and 0.85 M sodium hydroxide, pH 6.1) was added to each hydroxyproline sample and incubate at room temperature for 5 min. 100 μL of the DMAB reagent (2 g dimethylaminobenzaldehyde dissolved in 1.25 mL n-propanol and 2.75 mL perchloric acid) was added to each well and incubate for 90 min at 60 °C. The reaction was measure with a microplate reader (BioTek, Winooski, VT, USA) at 560 nm.

#### 4.5.3. Succinate-GloTM Hydroxylase Assay

Succinate-GloTM Hydroxylase assay was performed as the protocol described (Promega, V7991). Briefly, after the hCP4H1 enzyme reaction, 10 µL Succinate Detection Reagent I was added. The mixture was shanked for 30 s and incubated at room temperature for 60 min. 20 µL of Succinate Detection Reagent II was added to each well and incubated at room temperature for 10 min. The luminescence was measured with a plate-reading luminometer (BioTek).

### 4.6. High-Throughput Screening

All the chemicals (50 µM) were incubated with human C-P4H1 (200 nM) at least 30 min on ice. Then mix with peptide substrate (GlyProProGlyOEt, 500 μM), FeSO_4_ (50 μM), catalase (0.1 mg/mL), ascorbate (2 mM), and α-ketoglutarate (100 μM) to react 1 h at room temperature. The reaction volume was usually 10 µL. After the reaction, Succinate-GloTM Hydroxylase assay was conducted in the corresponding 96-well or 384-well plate. 50 µM DHB was set as a positive control in each plate.

### 4.7. Statistical Analysis

All the 3D culture assay images were acquired by Nikon microscope and were quantified with the Nikon analysis software. Results are reported as mean ± S.E.M; the significance of difference was assessed by independent Student’s *t*-test. *p* < 0.05 represents statistical significance and *p* < 0.01 represents sufficiently statistical significance. All reported *p* values were 2-tailed.
